# Soluble cluster of differentiation 14 levels elevated in bile from gallbladder cancer cases from Shanghai, China

**DOI:** 10.1038/s41598-021-92728-5

**Published:** 2021-06-28

**Authors:** Victoria L. Brun, Amanda F. Corbel, Ann W. Hsing, Troy J. Kemp, Alison L. Van Dyke, Allan Hildesheim, Bin Zhu, Yu-Tang Gao, Ligia A. Pinto, Jill Koshiol

**Affiliations:** 1 Partnership Development Office, Frederick National Laboratory for Cancer Research, Frederick, MD USA; 2 Stanford Prevention Research Center/Cancer Institute, Stanford University, Stanford, CA USA; 3 Vaccine, Immunity and Cancer Program, Frederick National Laboratory for Cancer Research, Frederick, MD USA; 4 Division of Cancer Epidemiology and Genetics, National Cancer Institute, Rockville, MD USA; 5 Department of Epidemiology, Shanghai Cancer Institute, Shanghai, China

**Keywords:** Biomarkers, Epidemiology, Cancer

## Abstract

Elevated systemic levels of soluble cluster of differentiation 14 (sCD14) have been associated with gallbladder cancer (GBC), but the association with sCD14 levels within the gallbladder has not been investigated. Here, we evaluated sCD14 in the bile of 41 GBC cases and 117 gallstone controls with data on 65 bile inflammation markers. We examined the relationship between bile sCD14 levels and GBC using logistic regression and stratified the analysis by stage. We included GBC-associated inflammatory biomarkers in the model to evaluate the influence of local inflammation. Bile sCD14 levels (third versus first tertile) were associated with GBC (adjusted odds ratio [OR]: 3.0, 95% confidence interval [CI]: 1.2–8.0). The association was equally strong for stage I/II (OR: 3.3, 95% CI: 0.9–15.6) and stage III/IV (OR: 3.2, 95% CI: 1.0–12.4) cancers. Including the GBC-associated inflammatory markers in the model removed the association between bile sCD14 and GBC (OR: 1.0, 95% CI: 0.3–3.5). The findings suggest that immune activation within the gallbladder may be related to GBC development, and the effect of sCD14 is influenced by inflammation. Similar associations across tumor stages suggest that elevated bile sCD14 levels may reflect changes early in GBC pathogenesis. Associations between GBC and sCD14 levels in both bile and plasma suggest sCD14 could be a potential biomarker for GBC.

## Introduction

Gallbladder cancer (GBC) is an aggressive and lethal malignancy that is linked to recurrent or chronic inflammation^1^, but its etiology remains poorly characterized. Gallstones, which can induce a state of epithelial irritation and chronic inflammation, are a dominant risk factor for GBC and are present in 75–90% of GBC patients^2^. Regular use of aspirin, a non-steroidal anti-inflammatory drug, has been associated with reduced GBC risk^2,3^ and improved survival^4^, underscoring the importance of inflammation in GBC etiology. However, few gallstone patients (0.3–3%) develop GBC, indicating that gallstones alone are insufficient for GBC development and additional factors are important in GBC development^2^.

Soluble cluster of differentiation 14 (sCD14) is a marker of immune activation^5^ and has primarily been studied in the context of HIV, and elevated sCD14 levels in plasma or serum are predictors of morbidity and mortality in HIV-infected patients^6,7^. It has also been associated with a variety of infectious and inflammatory conditions, including rheumatoid arthritis^8^, cystic fibrosis pulmonary exacerbations^9^, cardiovascular disease^10,11^, and hepatitis B or C virus infection^12,13^. Given its association with inflammation and the link between GBC and chronic inflammation, evaluating sCD14 in bile can provide insight into the relation between local immune activation and GBC.

In our earlier study of plasma sCD14 and GBC^14^, plasma sCD14 levels were five-fold higher in GBC cases compared to gallstone controls. However, plasma levels may not reflect the local immune response and inflammatory profile. Bile is a rich source of biomarkers and more closely reflects the local inflammatory response in the gallbladder and biliary tract. To better understand the processes occurring within the gallbladder, we examined bile sCD14 levels from GBC patients and gallstone patients without GBC. We also evaluated the relationships between bile sCD14 and other inflammation-related biomarkers measured in bile previously^15,16^ to investigate sCD14 in the larger context of the local inflammatory response.

## Materials and methods

### Study population and data collection

The biological specimens and data were obtained from the Shanghai Biliary Tract Cancer Study, which enrolled participants from June 1997 to May 2001. The study has been previously described^14–17^. It utilized a rapid reporting system to ascertain newly diagnosed GBC patients from 42 hospitals in urban Shanghai, China. Gallstone controls without GBC were recruited at the same hospital as the GBC cases and were frequency matched by 5-year age group and sex. All cases and controls were between the ages of 34 and 74, were permanent residents of urban Shanghai, and had no prior history of cancer except non-melanoma skin cancer. A total of 368 GBC cases and 774 gallstone controls were enrolled, and participation rates for eligible GBC cases and gallstone controls were greater than 90% and 95%, respectively. The U.S. National Cancer Institute and Shanghai Cancer institutional review boards approved the study. All participants provided written informed consent, and all research was performed in accordance with relevant guidelines and regulations.

For our analysis, we included all GBC cases for which paired bile and plasma samples were available (n = 41, 11%) and 117 (~ 3:1) randomly selected gallstone controls with available bile and plasma samples^15^. Bile was aspirated from the gallbladder under aseptic conditions via syringe.

### Detection of plasma and bile sCD14

The methods used to assess sCD14 in plasma or bile have been previously described^14,16^. Bile sCD14 levels were quantified (pg/mL) using the Quantikine enzyme-linked immunosorbent assay (ELISA) kit (R&D Systems Cat# DC140) in adherence to the manufacturer’s protocol. Bile samples were diluted 1:40 with a few exceptions that exceeded the highest detectable level and required further dilution. Samples were assayed in duplicate, and sample concentrations were calculated via a four-parameter logistic fit curve using the SoftMax Pro 6.3 (Molecular Devices, LLC) program. The laboratory controls provided in the kit were assayed in duplicate and used for all assays.

For bile sCD14 levels above the upper limit of quantification, the levels were assigned the upper limit (1,803,336 pg/mL). If the levels were below the lower limit of quantification, they were assigned half the lower limit (2,500 pg/mL).

### Inflammation markers

We also examined the correlation between 65 bile biomarkers associated with inflammation (Supplementary Table S3). Biomarkers included acute-phase proteins, pro- and anti-inflammatory cytokines, chemokines, growth factor receptors, and angiogenesis factors previously examined in bile from this study population^15,16^. Six markers were identified as a priori markers based on previous work that demonstrated an association between the markers and early-stage GBC: soluble tumor necrosis factor receptor 2 (sTNFR2), sTNFR1, CC motif ligand 20 (CCL20), vascular cell adhesion molecule 1 (VCAM-1), IL-16, and granulocyte colony-stimulating factor (G-CSF)^18^.

### Statistical analysis

All analyses were performed using SAS 9.4 (Cary, North Carolina). Characteristics between GBC cases and gallstone controls were compared using univariate analysis with Fisher’s exact test for categorical variables. For continuous variables, we used Wilcoxon rank sum tests for variables with a skewed distribution and *t*-tests for normally distributed variables. Normality assumptions were tested with a quantile–quantile plot and the Kolmogorov–Smirnov test. To investigate whether our study population was representative of the original study population, we performed univariate analyses with the chi-square test to compare the GBC cases and gallstone controls used in this study to the remaining GBC cases and gallstone controls from the Shanghai Biliary Tract Cancer Study. The sCD14 values for both plasma and bile were categorized into tertiles based on the gallstone controls. The first tertile served as the referent for the subsequent analyses.

We used unconditional logistic regression to examine the relationship between GBC and bile sCD14 levels. A backward regression analysis was used to evaluate potential confounders, including sex (male/female), age (≤ 54, 55–65, ≥ 66), body mass index (BMI) (underweight, normal, overweight/obese), ever smoking, and batch. We assessed whether any potential covariates changed the odds ratio (OR) for bile sCD14 more than 10%, and none did. Consequently, only sex and age were retained in the model, as the original study design frequency matched on these factors.

The correlation between sCD14 plasma and bile was assessed using Spearman rank correlation in two ways: (1) inclusive of all samples, which may be influenced by detection versus non-detection, and (2) limited to specimens with detectable values only, to focus on correlations between actual levels. We computed the correlation when controlling for sex and plasma batch and for GBC cases and controls separately. We also calculated the Spearman rank correlation between sCD14 and the 65 inflammatory biomarkers. All tests used two-sided p-values for significance.

### GBC-associated inflammatory biomarkers

To reduce the dimensionality of the inflammatory marker data, we performed cluster analysis and stepwise logistic regression. First, we identified the bile inflammatory biomarkers associated with GBC at *p* < 0.05 when categorized as above or below the median. We performed a cluster analysis with the resulting 25 biomarkers, which yielded 10 clusters (Supplementary Table S1). To represent each cluster, we selected the biomarker with the strongest association with GBC. We then performed a stepwise logistic regression for the 10 continuous inflammation markers, bile sCD14, age, and sex with GBC as the outcome. We used a significance level of 0.1 to be added to the model and 0.05 to be retained. After stepwise logistic regression, only GRO, which is a pan-specific marker that detects C-X-C motif ligand 1,2,3 (CXCL1,2,3), and interleukin (IL)-33 remained associated with GBC. To evaluate the impact of inflammation on the association between bile sCD14 and GBC, we included the selected two biomarkers in the model as continuous variables with sCD14, age, and sex. We investigated other approaches to modeling and saw comparable results.

### Ethics approval and consent

The U.S. National Cancer Institute and Shanghai Cancer institutional review boards approved the study. All study participants provided informed written consent prior to study enrollment. All research was performed in accordance with relevant guidelines and regulations.

## Results

### Descriptive characteristics

The GBC cases and gallstone controls were similar in terms of age, sex, education, diabetes, BMI, smoking, and drinking, but GBC cases tended to be slightly older (median 68 and 66, respectively) and were more likely to be female (73% versus 68%) (Table 1). Thirty-four (83%) of the GBC cases also had gallstones at the time of surgery.

**Table 1:** Participant characteristics by GBC case and gallstone control status.

	GBC Cases	Gallstone Controls
N (%)	41 (25.9)	117 (74.1)
**Sex**		
Male	11 (26.8)	38 (32.5)
Female	30 (73.2)	79 (67.5)
**Age at interview**		
≤ 54	4 (9.8)	18 (15.4)
55–65	13 (31.7)	32 (27.4)
≥ 66	24 (58.5)	67 (57.3)
**Highest level of education**		
None	15 (36.6)	38 (32.5)
Primary/junior middle	12 (29.3)	28 (23.9)
Senior middle	5 (12.2)	31 (26.5)
College/university	9 (22.0)	20 (17.1)
**Diabetes mellitus**		
Yes	4 (9.8)	17 (14.5)
No	37 (90.2)	100 (85.5)
Cigarette smoking pack-years among smokers: mean ± SD	15.2 ± 14.7	26.1.0 ± 19.0
**Ever-smoking status**^†^		
Never	32 (78.1)	88 (75.9)
Current/Former	9 (21.9)	28 (24.1)
BMI 5-years ago: mean ± SD	24.1 ± 3.1	24.4 ± 3.0
**Ever-drinker status**		
Yes	8 (19.5)	22 (18.8)
No	33 (80.5)	95 (81.2)
**Gallstone status**		
No gallstones	7 (17.1)	0 (0)
Gallstones	34 (82.9)	117 (100)

^†^N < 117 for the gallstone controls, because of missing data.

The GBC cases included in the present study were similar to the remaining GBC cases in the Shanghai Biliary Tract Cancer Study in respect to sex, age, diabetes, stage, and two of the three TNM staging system variables (Supplementary Table S2), but they differed on the presence of distant metastases. Seven cases (18%) in the present study had distant metastases, compared to 101 cases (35.3%) from the original study (*p* = 0.03). However, 41 (11%) of the cases were missing values for this variable. For the gallstone controls, the two groups were similar in respect to sex and diabetes, but differed by age (*p* < 0.0001), with those in the present study being older than the remaining controls (median 66 and 60, respectively).

### Correlation in bile and plasma

In those with bile sCD14 detectable levels (37 cases [90.2%] and 101 controls [86.3%]), the correlation between sCD14 in bile and plasma was not significant after controlling for plasma batch and sex (*r*_s_ = 0.1, *p* = 0.1). There were differences between cases and controls. Restricted to patients with detectable sCD14 bile levels, there was a suggestion of a modest correlation between bile sCD14 and plasma sCD14 for GBC cases (*r*_s_ = 0.2, *p* = 0.2) but not for gallstone controls (*r*_s_ = 0.05, p = 0.6). Among all patients, including those with undetectable levels, there was no evidence of correlation (*r*_s_ = 0.006, *p* = 1.0, for GBC cases and *r*_s_ = 0.09, *p* = 0.3, for gallstone controls).

The median bile sCD14 level was 108,970 pg/mL bile (interquartile range [IQR]: 25,407–235,360 pg/mL) among GBC cases and 41,277 pg/mL bile (IQR: 11,898–96,937 pg/mL) among gallstone controls. These levels were somewhat lower than those observed in plasma where the median was 1,235,112 pg/mL of plasma (IQR: 938,392–1,791,512 pg/mL) for cases and 954,096 pg/mL of plasma (IQR: 814,200–1,148,576 pg/mL) for controls.

### Association between sCD14 levels and GBC

Bile sCD14 levels (third versus first tertile) were associated with GBC relative to the gallstone controls (adjusted odds ratio [OR]: 3.0, 95% confidence interval [CI]: 1.2–8.0) (Table 2). The association increased slightly after restricting to just GBC cases with gallstones (OR: 3.5, 95% CI: 1.3–10.7). We stratified the cases by tumor stage and found similar ORs among stage I/II tumors (n = 15) and stage III/IV tumors (n = 24). The adjusted OR for bile sCD14 levels (third versus first tertile) for stage I/II was 3.3 (95% CI: 0.9–15.6) and for stage III/IV was 3.2 (95% CI: 1.0–12.4).

**Table 2:** Observed bile sCD14 levels by GBC case and gallstone control status.

	Controls (n = 117)	Cases (n = 41)		Stage I or II (n = 15)		Stage III or IV (n = 24)	
	n (%)	n (%)	OR^‡^ (95% CI)	n (%)	OR^‡^ (95% CI)	n (%)	OR^‡^ (95% CI)
**Tertiles**^†^							
1st	38 (32.5)	8 (19.5)	1	3 (20.0)	1	4 (16.7)	1
2nd	39 (33.3)	9 (22.0)	1.06 (0.4–3.1)	1 (6.7)	0.3 (0.01–2.6)	8 (33.3)	1.9 (0.5–7.7)
3rd	40 (34.2)	24 (58.5)	3.01 (1.2–8.0)	11 (73.3)	3.3 (0.9–15.6)	12 (50.0)	3.2 (1.0–12.4)
*p* value		*0.03*		*0.02*		*0.2*	
*p* trend			*0.01*		*0.04*		*0.06*

^†^Bile sCD14 cutoffs among GS controls: tertiles < 16,676 pg/mL (first), ≥ 16,676 and < 81,906.4 pg/mL (second), ≥ 81,906.4 pg/mL (third).

^‡^Odds ratio adjusted for sex and age.

p value: Fisher’s exact test; p trend: Wald test.

### Inflammatory biomarkers

Of the six a priori inflammation markers associated with early stage GBC, three had significant correlations with bile sCD14 levels when limited to those with detectable bile sCD14 levels: VCAM-1 (*r*_s_ = 0.4, *p* < 0.0001), CCL20 (*r*_s_ = 0.3, *p* = 0.003), and IL-16 (*r*_s_ = 0.2, *p* = 0.01) (Supplementary Table S3). Of the 59 other markers, two were strongly correlated with bile sCD14 levels when limited to those with detectable levels: complement factor D (adipsin) (*r*_s_ = 0.5, *p* < 0.0001) and soluble glycoprotein 130 (sGP130) (*r*_s_ = 0.5, *p* < 0.0001). Without limiting to those with detectable sCD14 levels, five markers were strongly correlated: adipsin (*r*_s_ = 0.6), sGP130 (*r*_s_ = 0.6), soluble interleukin receptor 6 (sIL-6R) (*r*_s_ = 0.5), C–C motif ligand 2 (CCL2) (*r*_s_ = 0.5), and C-X-C motif ligand 6 (CXCL6) (*r*_s_ = 0.5). These associations were all statistically significant (*p* < 0.0001) even after Bonferroni correction for multiple comparisons. After adjusting for the two bile markers retained after cluster analysis and stepwise logistic regression (CXCL1,2,3 and IL-33), the OR for all GBC cases combined and bile sCD14 levels (third versus first tertile) was reduced to 1.0 (95% CI: 0.3–3.5) (Table 3). For stage I/II, the OR decreased to 1.0 (95% CI: 0.2–5.9), and for stage III/IV it also decreased to 1.0 (95% CI: 0.1–7.1).

**Table 3:** Observed bile sCD14 levels by GBC case and gallstone control status adjusted by GBC-associated inflammatory markers.

	Cases	
	OR^‡^ (95% CI)	*p* trend/*p* value
**sCD14 Tertiles**^**†**^		*1.0*
1st	1	
2nd	0.8 (0.2–3.1)	
3rd	1.0 (0.3–3.5)	
CXCL1,2,3–100 pg/mL	1.1 (1.0–1.1)	< *0.0001*
IL-33–10 pg/mL	0.9 (0.9–1.0)	*0.002*
**Age at interview**		*0.5*
≤ 54	1	
55–65	1.2 (0.2–6.4)	
≥ 66	1.5 (0.4–7.4)	
Sex (male)	0.9 (0.3–2.6)	*0.9*

^†^Bile sCD14 cutoffs among GS controls: tertiles < 16,676 pg/mL (first), ≥ 16,676 and < 81,906.4 pg/mL (second), ≥ 81,906.4 pg/mL (third).

^‡^Odds ratio is mutually adjusted.

p trend (sCD14 Tertiles and Age) and p value (CXCL1,2,3, IL-33, CCL17, and sex): Wald test.

## Discussion

We examined bile sCD14, for the first time to our knowledge, and its association with GBC compared to gallstones, as well as its association with other inflammatory biomarkers. We found that elevated bile sCD14 levels were associated with a three-fold increased GBC risk, and this association held across tumor stages, suggesting sCD14 may be relevant in early GBC development and not simply reflective of inflammatory responses associated with late-stage GBC. We did not see a strong correlation between bile and plasma sCD14 levels, which suggests differences between the local and systemic immune response. Despite the poor correlation between bile and serum levels of sCD14, sCD14 levels were elevated in both bile and serum from GBC cases, suggesting that this protein might be a useful biomarker for GBC. When we accounted for inflammation, as captured by CXCL1,2,3 and IL-33, the association between sCD14 in bile and GBC disappeared, suggesting that inflammation could be affecting the association between sCD14 and GBC.

The two selected GBC-associated inflammation markers that removed the association between bile sCD14 and GBC have both been associated with cancer, and specifically, with changes in the tumor microenvironment in previous studies. IL-33 is expressed at high levels in the tumor microenvironment^19^, and during cancer progression, IL-33 is upregulated in the tumor stroma and serum, where it facilitates immune suppression via T-regulatory cells (T-regs) and myeloid-derived suppressor cells^19,20^, although its effects depend on the level of expression^21^. CXCL1,2,3 chemokines are also involved in recruiting immune cells such as myeloid cells and tumor-associated neutrophils to the tumor microenvironment and have been associated with increased tumor survival and metastasis^22–25^. Higher levels of CXCL1 in gastric cancer have been associated with tumor progression and reduced patient survival^26^.

The three a priori markers significantly correlated with sCD14 levels have been reported to be associated with cancer. CCL20, which is known to contribute to the progression of many cancers, mediates T-reg infiltration into the tumor microenvironment and facilitates cancer progression and poor prognosis in hepatocellular carcinoma (HCC) and colorectal cancer patients^27,28^. Il-16 regulates T cell growth and has been associated with different type of cancers, but its role appears to vary by cancer type^29^. Increasing evidence indicates the VCAM-1 has a role in tumor angiogenesis and metastasis across multiple cancer types^30^.

We found five additional biomarkers to be associated with bile sCD14 levels. CCL2 is associated with both tumor-promoting and tumor-suppressing activities, activating pro-tumor macrophages and enhancing anti-tumor neutrophil activity^31,32^. CXCL6 is elevated in HCC tumors and is associated with metastasis and poor prognosis^33^. sGP130 inhibits IL-6 trans-signaling, which is enabled by sIL-6R and is known to facilitate inflammation and have a role in cancer. sGP130 can interfere with anti-inflammatory classical signaling at high concentrations^34,35^. Adipsin is a key component of the tumor microenvironment in breast cancer^36^. Taken together, these associations, and the associations between these inflammatory markers and sCD14 in the present study, suggest that sCD14 may be involved in inflammatory changes in the microenvironment that lead to GBC development, but further studies are needed to interrogate the precise mechanisms involved.

The lack of correlation between sCD14 in bile and plasma is consistent with our previous observations for immune-related markers^15^. However, both circulating sCD14 and bile sCD14 were associated with GBC, suggesting that the local association with GBC is reflected systemically, despite the lack of strong correlation. In addition, just as we found that inflammation markers in the bile accounted for the association between bile sCD14 and GBC, circulating inflammation markers strongly attenuated the association between plasma sCD14 and GBC^14^, supporting the mirroring of associations between bile and blood.

The exact mechanism for the elevation of sCD14 in GBC is unknown. sCD14 is a co-receptor for lipopolysaccharide, which is found on the outer membrane of gram-negative bacteria. Several studies have linked gram-negative bacteria, including *Helicobacter* species and *Salmonella enterica* serovariant Typhi, to GBC^37–40^, and sCD14 could be an indicator of a response to bacterial infection. However, other toll-like receptor ligands, including flagellin and CpG oligodeoxynucleotides, can also induce sCD14 release^5,41^. In future studies, 16S rRNA gene sequencing could identify the presence of bacteria and help elucidate whether gram-negative bacteria are inducing sCD14 production in GBC. Studies of monocytes and sCD14 in GBC are also merited.

Several previous studies have linked elevated sCD14 levels in blood to incidence of various cancers, including glioma^42^, liver cancer^43–45^, and epithelial ovarian cancer^46^. Glioma was associated with elevated serum sCD14 levels versus heathy controls, with an OR of 3.94 (95% CI 2.98–5.21) for the highest versus lowest quartile^42^, which is similar to the OR for the present study. In patients with chronic HBV infection, serum sCD14 levels were significantly elevated in HCC compared to healthy controls (OR: 1.314, *p* < 0.001) and enabled discrimination of HCC from other HBV-related non-HCC diseases (OR: 2.145, *p* < 0.001)^45^. In our previous study of sCD14 plasma levels for GBC cases versus gallstone controls, the OR was 5.41 (95% CI 2.0–16.75) for the first versus third tertiles, higher than what was found for bile^14^.

Previous studies also provide some evidence of an association between sCD14 and cancer progression. Patients with stage III/IV epithelial ovarian cancer had higher sCD14 serum levels than patients with stage I cancer (*p* = 0.005)^46^. In patients with HBV infection, serum sCD14 levels were predictive of overall survival of HCC patients (hazard ratio: 2.54, 95% CI:1.169–5.54, *p* = 0.02) and were significantly decreased post liver resection for HCC (*p* < 0.001)^45^. However, in the study on glioma patients, sCD14 serum levels were not found to differ by resection, medication, or radiation^42^. Another study found that sCD14 serum levels were able to distinguish HCC from liver cirrhosis, and sCD14 was proposed to have potential diagnostic value for early detection of HCC in combination with alpha-fetoprotein^44^.

In addition, elevated plasma sCD14 levels have been correlated with poor prognosis for primary biliary cholangitis^47^, a risk factor for hepatobiliary cancers, particularly HCC^48–50^. As the present study showed different associations with bile sCD14 for GBC cases compared to gallstone controls, similar to the previous results for plasma sCD14^14^, it supports the potential value of circulating sCD14 in identifying risk of GBC among those with gallstones, similar to HCC risk among those with cirrhosis. Early detection is particularly important in high-risk regions, such as Chile, which has the highest GBC incidence rate and mortality rate^51^. Because bile sCD14 was shown to be associated with stage I/II cancers, it offers potential for identifying GBC early, when it is more readily treatable. Taken together, these findings suggest that circulating sCD14 may have value as a biomarker for detecting cancer at an early stage as part of a multi-marker approach, and its role should be further investigated.

This study has several limitations. Given the cross-section study design, it is unclear whether the elevated sCD14 levels reflect cancer development or a response to the cancer. In addition, the study was conducted in a single geographic region, so it may not be representative of other populations. Also, because of the difficulty in obtaining bile, no bile samples were available from healthy donors for comparison. However, this limitation can also be considered a strength; using gallstone patients as controls enabled us to look beyond the immune response caused by the gallstones themselves. Because most GBC patients have gallstones, this distinction is valuable. The small sample size of GBC cases (n = 41) may have also yielded imprecise estimates. With 41 cases and 117 controls, the power for detection of the third tertile versus first tertile with an odds ratio of 3 is 66%. These cases were a small subset of the original GBC study population but were similar to those not included except with respect to the presence of distant metastases. In addition, we made multiple comparisons with inflammation markers. Although we corrected for multiple comparisons testing, some results may have been due to chance. Still, the magnitude of the estimate for sCD14 suggests an association worthy of further evaluation.

The study also has several notable strengths. To our knowledge, it is the first study to examine sCD14 in bile, and as such, it offers novel insights into the local immune response in GBC. In addition, we were able to explore the relationship between sCD14 and immune-related biomarkers in bile. We were also able to take advantage of the well-characterized epidemiological data to evaluate numerous potentially important confounders and covariates.

In summary, we found that elevated sCD14 bile levels are associated with an increased GBC risk, and that association appears to be influenced by inflammation. Although we did not find a strong correlation between bile and plasma sCD14 levels, the strong association observed between bile sCD14 and GBC is consistent with our previously observed association between elevated plasma sCD14 and GBC, suggesting that sCD14 is an important target for further research. Furthermore, the associations in both bile and plasma with GBC suggest that sCD14 is a potential biomarker for GBC. Future studies are merited to understand the biologic mechanisms involved in raising sCD14 levels in GBC. Studies evaluating evidence of infections would offer a useful next step to understanding whether bacteria or other mechanisms elevated sCD14 levels.

## Supplementary Information

Supplementary Information.

## Data Availability

The data that support the findings of this study are available on request from the corresponding author on reasonable request. The data are not publicly available due to privacy or ethical restrictions.
