# Supplementary material for: Soluble cluster of differentiation 14 levels elevated in bile from gallbladder cancer cases from Shanghai, China
Source: Sci Rep. 2021 Jun 28;11:13405. doi: 10.1038/s41598-021-92728-5 (PMC8239000; doi:10.1038/s41598-021-92728-5)
Supplement: Supplementary file 1 — Supplementary Information. [file 41598_2021_92728_MOESM1_ESM.pdf]

Title: Soluble cluster of differentiation 14 levels elevated in bile from gallbladder cancer cases from Shanghai, China

Authorship: Victoria L Brun, Amanda F Corbel, Ann W Hsing, Troy J Kemp, Alison L Van Dyke, Allan Hildesheim, Bin Zhu, Yu-Tang Gao, Ligia A Pinto, and Jill Koshiol

Table S1. Bile inflammatory biomarkers associated with GBC

Cluster	Biomarkers	GBC Cases		Gallstone Controls	
		Mean ± SD	Range	Mean ± SD	Range
1	CCL20†	3,214.5 ± 5,254.1	12.2–29,246.1	1,289.1 ± 4,341.9	12.2–41,734.9
	CCL21	3,320.0 ± 2,654.3	390.7–12,394.9	4,112.6 ± 2,516.4	390.7–14,096.0
	CCL27	141.6 ± 159.0	6.1–559.1	152.5 ± 121.6	6.1–484.7
	CCL24	4,070.2 ± 15,810.5	12.2–100,000.0	2,805.1 ± 2,988.0	12.2–13,340.2
2	IL-8†	28,307.6 ± 31,902.3	15.2–100,000.0	7,350.6 ± 20,148.1	3.2–100,000.0
	Lipocalin-2	1,044,979.6 ± 1,158,575.3	76,719.2–5,000,000.0	511,202.3 ± 611,377.4	5,051.1–5,000,000.0
	Resistin	355,292.3 ± 505,088.2	671.0–2,000,000.0	172,590.5 ± 424,039.8	140.1–2,000,000.0
3	CXCL1,2,3†	6,552.5 ± 6,029.6	80.0–32,189.5	1,534.8 ± 1,797.3	80.0–11,534.4
	CXCL9	26,428.6 ± 51,134.5	61.1–245,995.3	6,789.7 ± 6,857.4	61.1–30,924.3
	CXCL5	10,455.8 ± 14,141.5	97.7–49,461.1	1,429.2 ± 2,930.3	97.7–21,777.2
4	IL-33†	83.5 ± 143.1	24.4–682.7	172.00 ±312.1	24.4– 2,401.5
	CCL8	123.2 ± 145.4	24.4–864.9	142.2 ± 101.7	24.4–711.6
	CCL13	498.4 ± 386.8	195.3–1,607.8	669.2 ± 535.6	195.3–3,360.9
5	PAI-1†	21,991.3 ± 45,806.0	260.6–264,540.7	8,858.0 ± 23,839.1	96.0–206,876.6
	Adipsin	414,733.1 ± 393,570.5	640.0–2,157,400.0	235,141.9 ± 227,465.8	640.0–1,015,500.0
	CCL2	16,675.1 ± 21,721.8	32.6–82,921.6	8,518.7 ± 13,812.4	16.0–100,000.0
6	sRAGE†	421.6 ± 687.9	244.2–3,666.0	702.1 ± 1,310.5	244.2–9,930.2
	sVEGFR1	22,095.3 ± 35,985.1	2,441.4–167,248.0	29,622.3 ± 40,933.4	2,441.4–255,091.5
	sILRII	17,428.5 ± 45,223.7	2,441.4–277,782.9	13,231.3 ± 11,265.8	2,441.4–60,323.5
7	CCL17†	16.7 ± 17.6	1.2–72.9	25.6 ± 30.1	1.2–276.5
	CCL22	656.3 ± 738.7	16.0–3,899.1	517.5 ± 715.5	16.0–6,799.7
8	CCL15†	7,365.5 ± 8,699.2	952.8–47,946.9	22,056.6 ± 38,196.6	61.1–263,785.8
	sTNFRI	6,619.8 ± 11,603.8	244.2–70,451.2	7,970.1 ± 8,745.0	244.2–55,001.9
9	sEGFR†	205,330.4 ± 637,678.5	2,441.4–3,918,600.0	35,028.8 ± 115,029.8	2441.4–1,035,600.0
10	SAA†	3,812,897.3 ± 15,705,842.8	3,000.0–100,000,000.0	623,390.4 ± 3,143,470.3	3,000.0–29,529,101.0

†Biomarker was included in the stepwise regression model to represent the cluster.

Table S2. Participant characteristics for the study GBC cases and gallstone controls and the remaining GBC cases and gallstone controls in the XXXX Study

	**Study GBC Cases**	**Remaining GBC Cases**	**Study Gallstone Controls**	**Remaining Gallstone Controls**
N (%)	41 (11%)	328 (89%)	117 (15%)	657 (85%)
Sex				
Male	11 (26.8)	88 (26.8)	38 (32.5)	225 (34.3)
Female	30 (73.2)	240 (73.2)	79 (67.5)	432 (65.8)
Age at interview‡				
≤ 54	4 (9.8)	45 (13.8)	18 (15.4)	227 (34.6)
55–65	13 (31.7)	98 (30.0)	32 (27.4)	227 (34.6)
≥66	24 (58.5)	184 (56.3)	67 (57.3)	203 (30.9)
Diabetes mellitus‡				
Yes	4 (9.8)	47 (14.4)	17 (14.5)	64 (9.7)
No	37 (90.2)	279 (85.6)	100 (85.5)	593 (90.3)
Stage (Grouped)†‡				
I/II	15 (38.5)	90 (28.0)		
III/IV	24 (61.5)	232 (72.1)		
Pathology				
Primary Tumor Stage†‡				
1	7 (18.0)	35 (11.3)		
2	10 (25.6)	77 (24.8)		
3	12 (30.8)	101 (32.5)		
4	10 (25.6)	98 (31.5)		
Lymph Node Spread†‡				
0	18 (45.0)	136 (43.5)		
1	19 (47.5)	162 (51.8)		
2	3 (7.5)	15 (4.8)		
Distant Metastasis†‡				
No	32 (82.1)	185 (64.9)		
Yes	7 (18.0)	101 (35.3)		

† N < 41 for the study GBC cases, because of missing data.
‡ N < 328 for the remaining GBC cases, because of missing data.

Table S3. Correlation between bile sCD14 and 65 inflammation-related biomarkers in gallstone controls

Biomarker	Correlation (Detectable only)	P value	Correlation (All)	P value	Median (pg/mL)
Adipsin	0.5	<.0001	0.6	<.0001	153,112.6
sGP130	0.5	<.0001	0.6	<.0001	30,496.0
sIL-6R	0.5	<.0001	0.5	<.0001	2,553.2
VCAM-1	0.4	<.0001	0.5	<.0001	77,333.3
PAI-1	0.3	0.0004	0.3	0.002	2,439.4
CXCL13	0.3	0.001	0.5	<.0001	101.3
CCL2	0.3	0.001	0.5	<.0001	5,418.8
CXCL6	0.3	0.002	0.5	<.0001	848.2
CCL27	0.3	0.002	0.4	<.0001	149.5
CCL20	0.3	0.003	0.4	<.0001	349.8
CXCL9	0.3	0.004	0.5	<.0001	5,276.0
Adiponectin	0.3	0.006	0.2	0.08	346,821.2
ICAM-1	0.3	0.006	0.5	<.0001	168,168.1
CXCL5	0.3	0.007	0.3	0.0009	618.2
CCL19	0.3	0.01	0.4	<.0001	815.6
CXCL1,2,3	0.3	0.01	0.4	<.0001	1,037.5
CCL17	0.2	0.01	0.4	<.0001	20.0
SAA	0.2	0.01	0.3	0.0009	32,940.9
IL-16	0.2	0.01	0.3	0.003	195.5
CXCL12	0.2	0.01	0.3	0.0002	3,680.7
CCL22	0.2	0.02	0.3	0.0002	324.2
sVEGFR1	0.2	0.05	0.3	0.0005	13,785.7
sEGFR	0.2	0.09	0.1	0.1	6,858.1
sCD40L	0.2	0.09	0.2	0.03	862.6
sTNFR2	0.2	0.1	0.2	0.02	4,126.8
CRP	0.2	0.1	0.04	0.7	191,708.1
Resistin	0.2	0.1	0.03	0.8	11,336.5
CCL11	0.2	0.1	0.2	0.01	393.4
CCL7	0.2	0.1	0.2	0.02	413.8
CCL24	0.2	0.1	0.3	0.0001	1,908.0
IFNa2	0.2	0.1	0.2	0.01	336.8
CXCL10	0.1	0.2	0.4	<.0001	1,998.4
TNF-a	0.1	0.2	0.2	0.05	41.4
CXCL11	0.1	0.2	0.3	0.002	115.1
CCL4	0.1	0.3	0.3	0.0004	743.5
IL-33	0.1	0.3	0.1	0.2	60.5
G-CSF	0.1	0.3	0.2	0.03	859.9
FGF-2	0.1	0.3	0.2	0.04	1,940.4
CCL21	0.1	0.3	0.3	0.004	3,888.6

VEGF	0.1	0.4	0.2	0.09	2,006.4
IFNg	0.1	0.4	0.2	0.1	99.4
CX3CL1	0.1	0.4	0.3	0.01	1,305.6
sIL-1RI	0.1	0.4	0.1	0.1	488.2
LIF	0.1	0.4	-0.1	0.2	212.0
CCL8	0.1	0.5	0.1	0.5	141.3
IL-12p40	0.1	0.5	0.1	0.6	80.0
IL-8	0.1	0.6	0.03	0.8	376.9
sIL-RII	0.1	0.6	0.1	0.3	11,245.5
STNFR1	0.1	0.6	-0.01	0.9	5,961.3
IL-11	0.05	0.6	-0.1	0.5	130.5
Lipocalin-2	0.04	0.7	-0.1	0.5	331,910.3
Flt3L	0.02	0.9	0.1	0.2	80.0
IL-29	0.02	0.9	0.04	0.7	1,406.1
sIL-4R	0.02	0.9	0.1	0.1	976.6
sVEGFR3	0.01	0.9	0.1	0.4	2,441.4
TRAIL	0.005	1.0	0.2	0.1	247.4
SCF	0.000	1.0	-0.1	0.4	12.2
sRAGE	-0.04	0.7	0.04	0.7	244.2
TSLP	-0.05	0.6	-0.1	0.2	12.2
sVEGFR2	-0.1	0.4	0.04	0.7	2,441.4
EGF	-0.1	0.4	-0.2	0.01	856.7
sCD30	-0.1	0.4	-0.02	0.8	488.2
CCL15	-0.1	0.4	-0.02	0.8	12,446.0
TPO	-0.1	0.3	-0.2	0.1	244.2
CCL13	-0.2	0.04	-0.3	0.004	586.8

Key

CCL – C-C motif ligand
CRP – C-reactive protein
CXCL – C-X-C motif ligand
EGF – epidermal growth factor
FGF-2 – fibroblast growth factor 2
Flt3L – FMS-like tyrosine kinase 3 ligand
G-CSF – granulocyte colony-stimulating factor
IFNA2 – interferon alpha 2
IFNg – interferon gamma
IL – interleukin
LIF – leukemia inhibitory factor
PAI-1 – plasminogen activator inhibitor 1
SAA - serum amyloid a
sCD40L – soluble CD40-ligand
SCF – stem cell factor
sEGFR – soluble epidermal growth factor receptor
sGP130 – soluble GP130
sIL-4R – soluble interleukin receptor 4
sIL-6R – soluble interleukin receptor 6
sIL-RII – soluble interleukin receptor 2
sRAGE – soluble receptor for advanced glycation end products
sTNFR – soluble tumor necrosis factor receptor
sVEGFR – soluble vascular endothelial growth factor receptor
TNF-a – tumor necrosis factor alpha
TPO – thrombopoietin
TRAIL – TNF-related apoptosis-inducing ligand
TSLP – thymic stromal lymphopoietin
VCAM-1 – vascular cell adhesion molecule 1
VEGF – vascular endothelial growth factor
